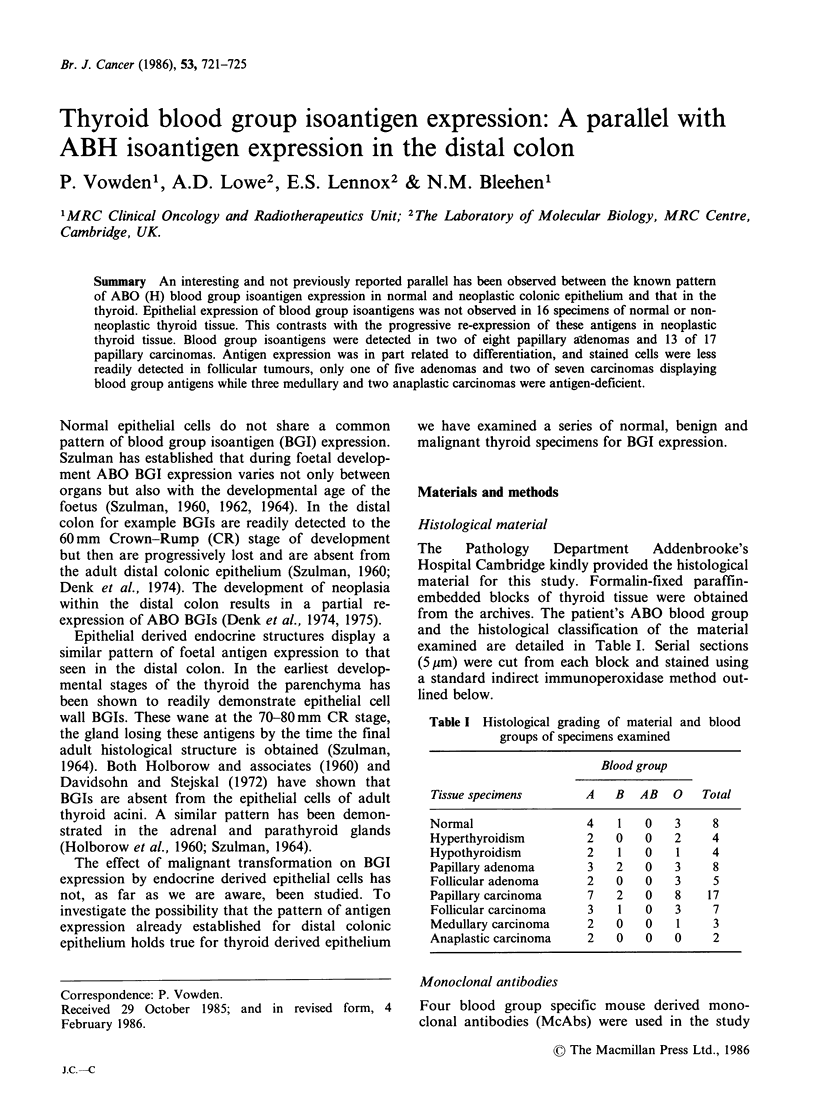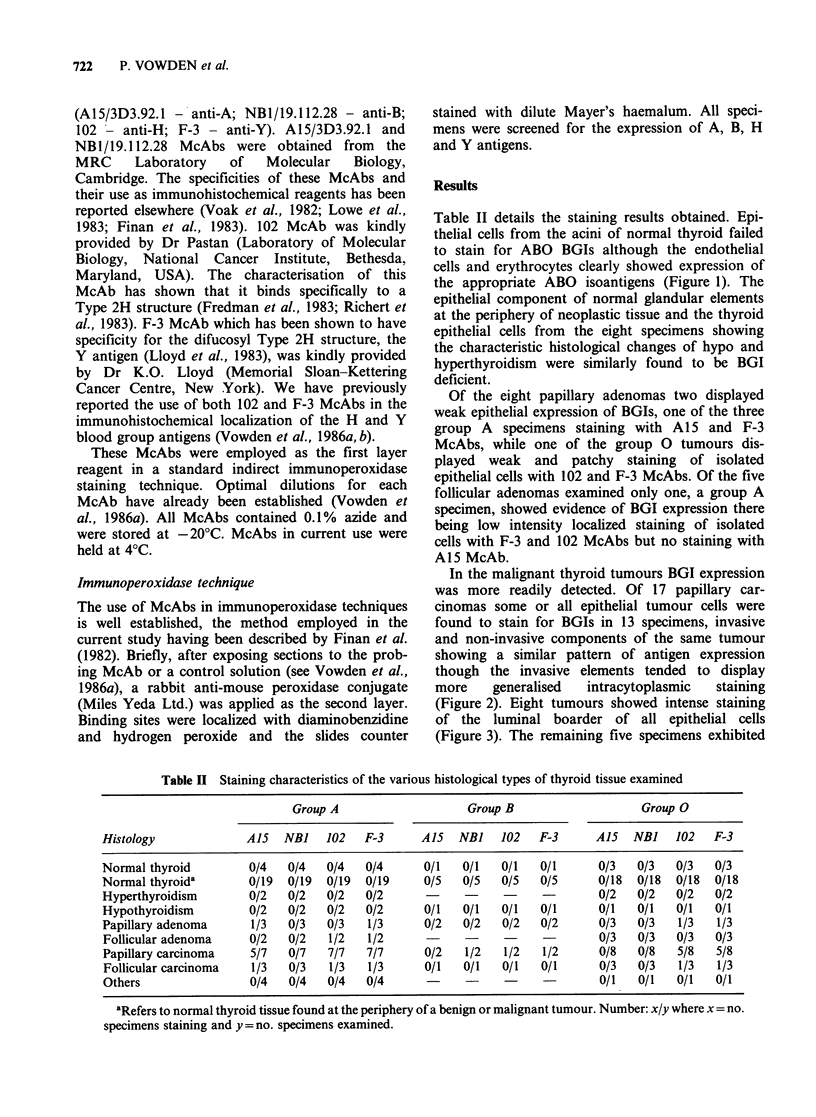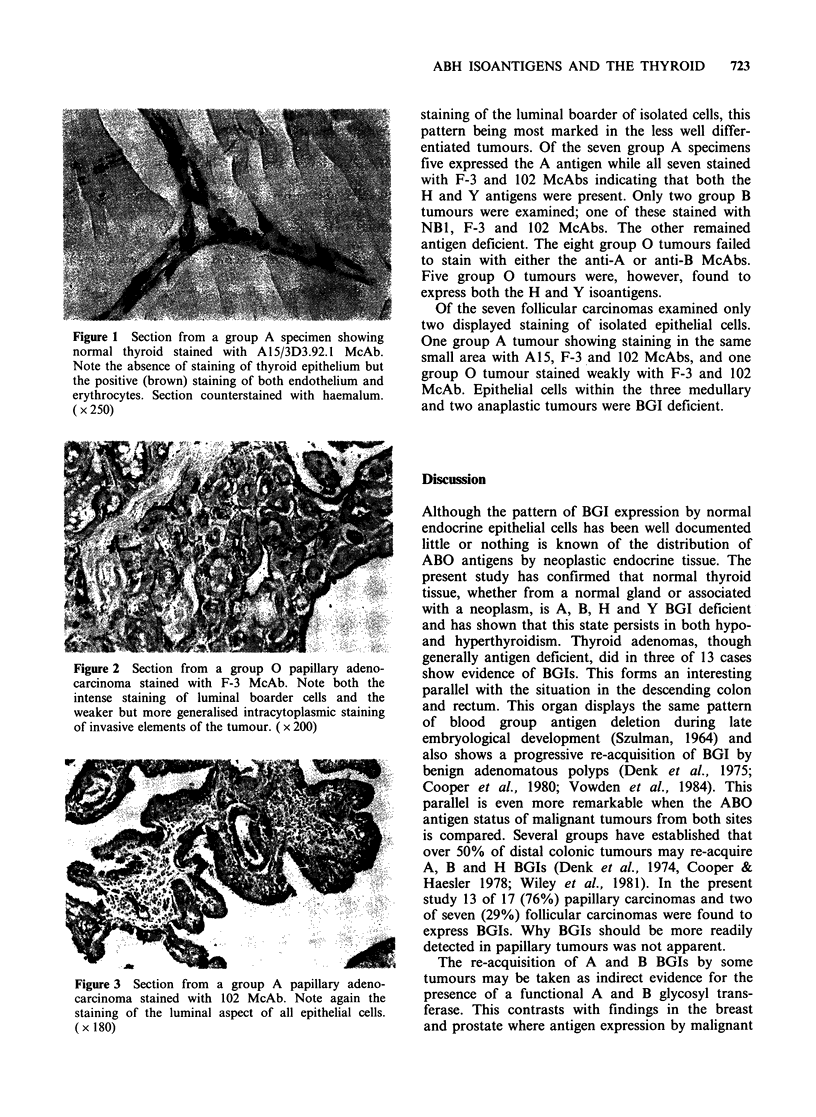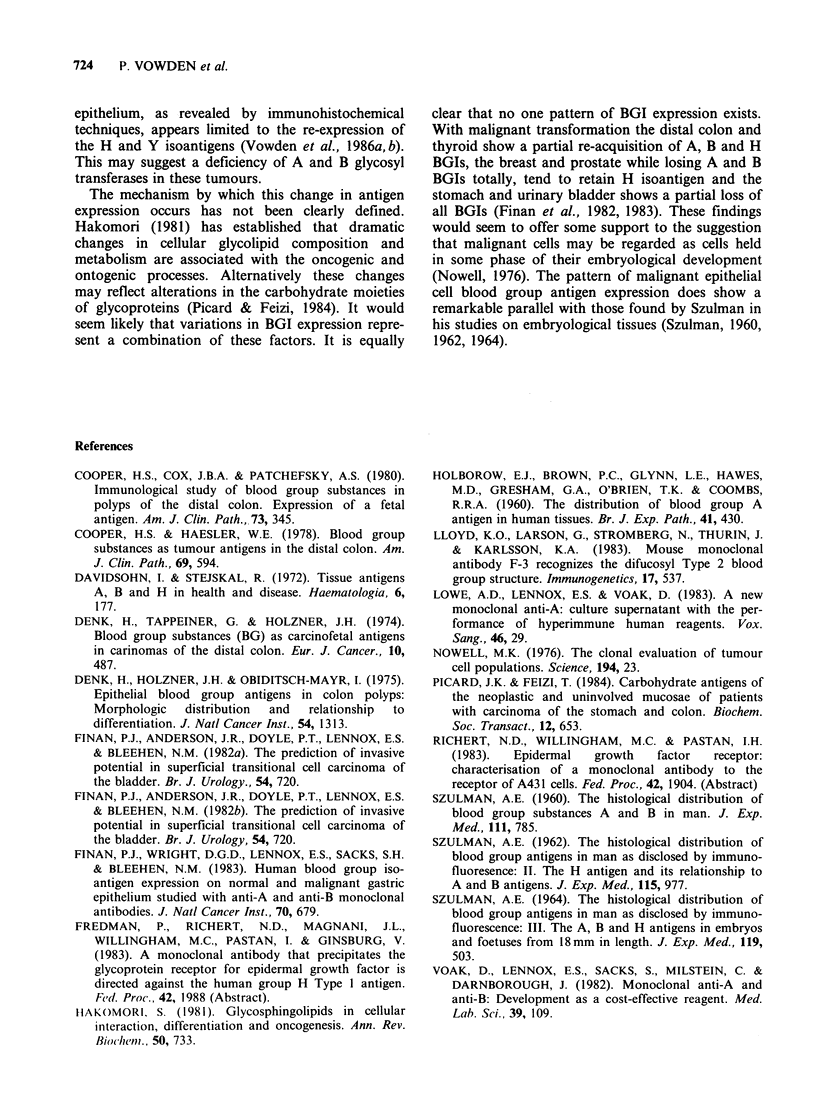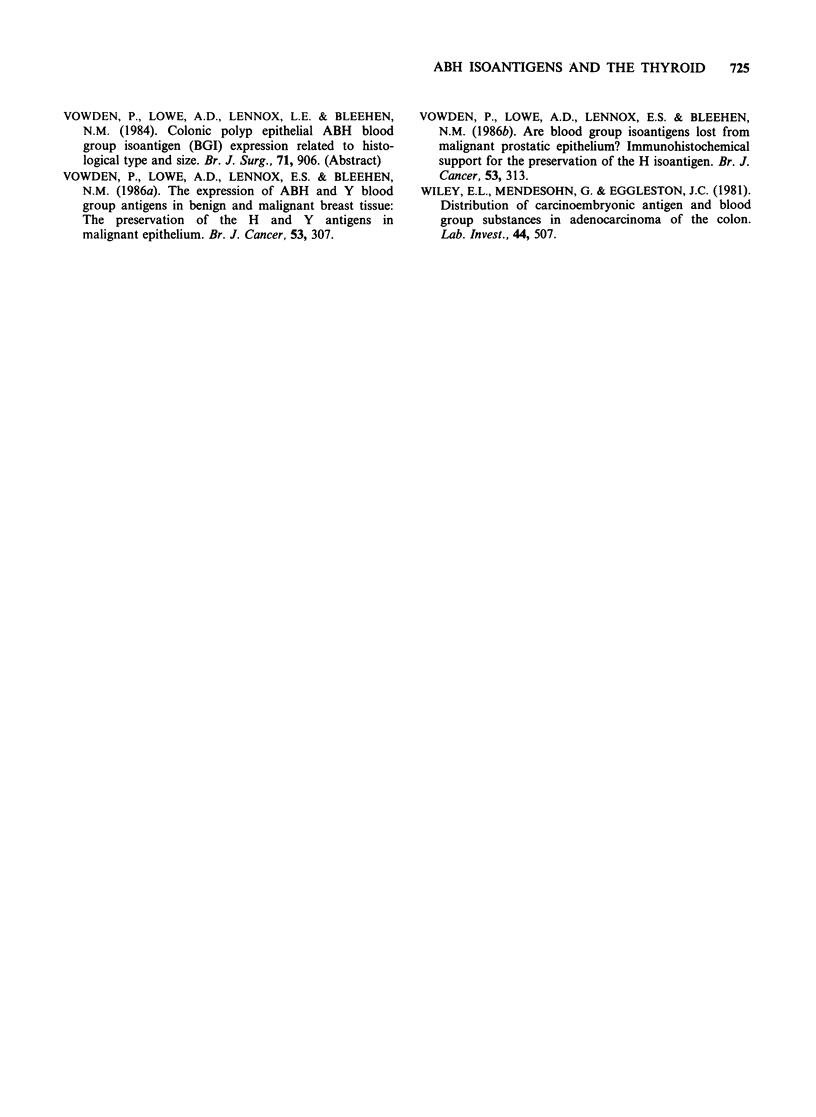# Thyroid blood group isoantigen expression: a parallel with ABH isoantigen expression in the distal colon.

**DOI:** 10.1038/bjc.1986.124

**Published:** 1986-06

**Authors:** P. Vowden, A. D. Lowe, E. S. Lennox, N. M. Bleehen

## Abstract

**Images:**


*Br. J. Cancer* (1986), **53**, 721–725

### Thyroid blood group isoantigen expression: A parallel with ABH isoantigen expression in the distal colon

P. Vowden[1], A.D. Lowe[2], E.S. Lennox[2] & N.M. Bleehen[1]

[1]*MRC Clinical Oncology and Radiotherapeutics Unit;* [2]*The Laboratory of Molecular Biology, MRC Centre, Cambridge, UK.*

**Summary** An interesting and not previously reported parallel has been observed between the known pattern of ABO (H) blood group isoantigen expression in normal and neoplastic colonic epithelium and that in the thyroid. Epithelial expression of blood group isoantigens was not observed in 16 specimens of normal or non-neoplastic thyroid tissue. This contrasts with the progressive re-expression of these antigens in neoplastic thyroid tissue. Blood group isoantigens were detected in two of eight papillary adenomas and 13 of 17 papillary carcinomas. Antigen expression was in part related to differentiation, and stained cells were less readily detected in follicular tumours, only one of five adenomas and two of seven carcinomas displaying blood group antigens while three medullary and two anaplastic carcinomas were antigen-deficient.

Normal epithelial cells do not share a common pattern of blood group isoantigen (BGI) expression. Szulman has established that during foetal development ABO BGI expression varies not only between organs but also with the developmental age of the foetus (Szulman, 1960, 1962, 1964). In the distal colon for example BGIs are readily detected to the 60 mm Crown–Rump (CR) stage of development but then are progressively lost and are absent from the adult distal colonic epithelium (Szulman, 1960; Denk *et al.,* 1974). The development of neoplasia within the distal colon results in a partial re-expression of ABO BGIs (Denk *et al.,* 1974, 1975).

Epithelial derived endocrine structures display a similar pattern of foetal antigen expression to that seen in the distal colon. In the earliest developmental stages of the thyroid the parenchyma has been shown to readily demonstrate epithelial cell wall BGIs. These wane at the 70–80 mm CR stage, the gland losing these antigens by the time the final adult histological structure is obtained (Szulman, 1964). Both Holborow and associates (1960) and Davidsohn and Stejskal (1972) have shown that BGIs are absent from the epithelial cells of adult thyroid acini. A similar pattern has been demonstrated in the adrenal and parathyroid glands (Holborow *et al.,* 1960; Szulman, 1964).

The effect of malignant transformation on BGI expression by endocrine derived epithelial cells has not, as far as we are aware, been studied. To investigate the possibility that the pattern of antigen expression already established for distal colonic epithelium holds true for thyroid derived epithelium we have examined a series of normal, benign and malignant thyroid specimens for BGI expression.

Correspondence: P. Vowden.
Received 29 October 1985; and in revised form, 4 February 1986.

### Materials and methods

### *Histological material*

The Pathology Department Addenbrooke's Hospital Cambridge kindly provided the histological material for this study. Formalin-fixed paraffin-embedded blocks of thyroid tissue were obtained from the archives. The patient's ABO blood group and the histological classification of the material examined are detailed in Table I. Serial sections (5 $\mu$m) were cut from each block and stained using a standard indirect immunoperoxidase method outlined below.

**Table I** Histological grading of material and blood groups of specimens examined

| | *Blood group* | | | | |
|---|---|---|---|---|---|
| *Tissue specimens* | *A* | *B* | *AB* | *O* | *Total* |
| Normal | 4 | 1 | 0 | 3 | 8 |
| Hyperthyroidism | 2 | 0 | 0 | 2 | 4 |
| Hypothyroidism | 2 | 1 | 0 | 1 | 4 |
| Papillary adenoma | 3 | 2 | 0 | 3 | 8 |
| Follicular adenoma | 2 | 0 | 0 | 3 | 5 |
| Papillary carcinoma | 7 | 2 | 0 | 8 | 17 |
| Follicular carcinoma | 3 | 1 | 0 | 3 | 7 |
| Medullary carcinoma | 2 | 0 | 0 | 1 | 3 |
| Anaplastic carcinoma | 2 | 0 | 0 | 0 | 2 |

### *Monoclonal antibodies*

Four blood group specific mouse derived monoclonal antibodies (McAbs) were used in the study

© The Macmillan Press Ltd., 1986

(A15/3D3.92.1 – anti-A; NB1/19.112.28 – anti-B; 102 – anti-H; F-3 – anti-Y). A15/3D3.92.1 and NB1/19.112.28 McAbs were obtained from the MRC Laboratory of Molecular Biology, Cambridge. The specificities of these McAbs and their use as immunohistochemical reagents has been reported elsewhere (Voak *et al.*, 1982; Lowe *et al.*, 1983; Finan *et al.*, 1983). 102 McAb was kindly provided by Dr Pastan (Laboratory of Molecular Biology, National Cancer Institute, Bethesda, Maryland, USA). The characterisation of this McAb has shown that it binds specifically to a Type 2H structure (Fredman *et al.*, 1983; Richert *et al.*, 1983). F-3 McAb which has been shown to have specificity for the difucosyl Type 2H structure, the Y antigen (Lloyd *et al.*, 1983), was kindly provided by Dr K.O. Lloyd (Memorial Sloan–Kettering Cancer Centre, New York). We have previously reported the use of both 102 and F-3 McAbs in the immunohistochemical localization of the H and Y blood group antigens (Vowden *et al.*, 1986*a, b*).

These McAbs were employed as the first layer reagent in a standard indirect immunoperoxidase staining technique. Optimal dilutions for each McAb have already been established (Vowden *et al.*, 1986*a*). All McAbs contained 0.1% azide and were stored at −20°C. McAbs in current use were held at 4°C.

*Immunoperoxidase technique*

The use of McAbs in immunoperoxidase techniques is well established, the method employed in the current study having been described by Finan *et al.* (1982). Briefly, after exposing sections to the probing McAb or a control solution (see Vowden *et al.*, 1986*a*), a rabbit anti-mouse peroxidase conjugate (Miles Yeda Ltd.) was applied as the second layer. Binding sites were localized with diaminobenzidine and hydrogen peroxide and the slides counter stained with dilute Mayer's haemalum. All specimens were screened for the expression of A, B, H and Y antigens.

### Results

Table II details the staining results obtained. Epithelial cells from the acini of normal thyroid failed to stain for ABO BGIs although the endothelial cells and erythrocytes clearly showed expression of the appropriate ABO isoantigens (Figure 1). The epithelial component of normal glandular elements at the periphery of neoplastic tissue and the thyroid epithelial cells from the eight specimens showing the characteristic histological changes of hypo and hyperthyroidism were similarly found to be BGI deficient.

Of the eight papillary adenomas two displayed weak epithelial expression of BGIs, one of the three group A specimens staining with A15 and F-3 McAbs, while one of the group O tumours displayed weak and patchy staining of isolated epithelial cells with 102 and F-3 McAbs. Of the five follicular adenomas examined only one, a group A specimen, showed evidence of BGI expression there being low intensity localized staining of isolated cells with F-3 and 102 McAbs but no staining with A15 McAb.

In the malignant thyroid tumours BGI expression was more readily detected. Of 17 papillary carcinomas some or all epithelial tumour cells were found to stain for BGIs in 13 specimens, invasive and non-invasive components of the same tumour showing a similar pattern of antigen expression though the invasive elements tended to display more generalised intracytoplasmic staining (Figure 2). Eight tumours showed intense staining of the luminal boarder of all epithelial cells (Figure 3). The remaining five specimens exhibited

**Table II** Staining characteristics of the various histological types of thyroid tissue examined

| | *Group A* | | | | *Group B* | | | | *Group O* | | | |
|---|---|---|---|---|---|---|---|---|---|---|---|---|
| *Histology* | *A15* | *NB1* | *102* | *F-3* | *A15* | *NB1* | *102* | *F-3* | *A15* | *NB1* | *102* | *F-3* |
| Normal thyroid | 0/4 | 0/4 | 0/4 | 0/4 | 0/1 | 0/1 | 0/1 | 0/1 | 0/3 | 0/3 | 0/3 | 0/3 |
| Normal thyroid[a] | 0/19 | 0/19 | 0/19 | 0/19 | 0/5 | 0/5 | 0/5 | 0/5 | 0/18 | 0/18 | 0/18 | 0/18 |
| Hyperthyroidism | 0/2 | 0/2 | 0/2 | 0/2 | — | — | — | — | 0/2 | 0/2 | 0/2 | 0/2 |
| Hypothyroidism | 0/2 | 0/2 | 0/2 | 0/2 | 0/1 | 0/1 | 0/1 | 0/1 | 0/1 | 0/1 | 0/1 | 0/1 |
| Papillary adenoma | 1/3 | 0/3 | 0/3 | 1/3 | 0/2 | 0/2 | 0/2 | 0/2 | 0/3 | 0/3 | 1/3 | 1/3 |
| Follicular adenoma | 0/2 | 0/2 | 1/2 | 1/2 | — | — | — | — | 0/3 | 0/3 | 0/3 | 0/3 |
| Papillary carcinoma | 5/7 | 0/7 | 7/7 | 7/7 | 0/2 | 1/2 | 1/2 | 1/2 | 0/8 | 0/8 | 5/8 | 5/8 |
| Follicular carcinoma | 1/3 | 0/3 | 1/3 | 1/3 | 0/1 | 0/1 | 0/1 | 0/1 | 0/3 | 0/3 | 1/3 | 1/3 |
| Others | 0/4 | 0/4 | 0/4 | 0/4 | — | — | — | — | 0/1 | 0/1 | 0/1 | 0/1 |

[a]Refers to normal thyroid tissue found at the periphery of a benign or malignant tumour. Number: $x/y$ where $x$ = no. specimens staining and $y$ = no. specimens examined.

**Figure 1** Section from a group A specimen showing normal thyroid stained with A15/3D3.92.1 McAb. Note the absence of staining of thyroid epithelium but the positive (brown) staining of both endothelium and erythrocytes. Section counterstained with haemalum. ( × 250)

**Figure 2** Section from a group O papillary adenocarcinoma stained with F-3 McAb. Note both the intense staining of luminal boarder cells and the weaker but more generalised intracytoplasmic staining of invasive elements of the tumour. ( × 200)

**Figure 3** Section from a group A papillary adenocarcinoma stained with 102 McAb. Note again the staining of the luminal aspect of all epithelial cells. ( × 180)

staining of the luminal boarder of isolated cells, this pattern being most marked in the less well differentiated tumours. Of the seven group A specimens five expressed the A antigen while all seven stained with F-3 and 102 McAbs indicating that both the H and Y antigens were present. Only two group B tumours were examined; one of these stained with NB1, F-3 and 102 McAbs. The other remained antigen deficient. The eight group O tumours failed to stain with either the anti-A or anti-B McAbs. Five group O tumours were, however, found to express both the H and Y isoantigens.

Of the seven follicular carcinomas examined only two displayed staining of isolated epithelial cells. One group A tumour showing staining in the same small area with A15, F-3 and 102 McAbs, and one group O tumour stained weakly with F-3 and 102 McAb. Epithelial cells within the three medullary and two anaplastic tumours were BGI deficient.

### Discussion

Although the pattern of BGI expression by normal endocrine epithelial cells has been well documented little or nothing is known of the distribution of ABO antigens by neoplastic endocrine tissue. The present study has confirmed that normal thyroid tissue, whether from a normal gland or associated with a neoplasm, is A, B, H and Y BGI deficient and has shown that this state persists in both hypo- and hyperthyroidism. Thyroid adenomas, though generally antigen deficient, did in three of 13 cases show evidence of BGIs. This forms an interesting parallel with the situation in the descending colon and rectum. This organ displays the same pattern of blood group antigen deletion during late embryological development (Szulman, 1964) and also shows a progressive re-acquisition of BGI by benign adenomatous polyps (Denk *et al.*, 1975; Cooper *et al.*, 1980; Vowden *et al.*, 1984). This parallel is even more remarkable when the ABO antigen status of malignant tumours from both sites is compared. Several groups have established that over 50% of distal colonic tumours may re-acquire A, B and H BGIs (Denk *et al.*, 1974, Cooper & Haesler 1978; Wiley *et al.*, 1981). In the present study 13 of 17 (76%) papillary carcinomas and two of seven (29%) follicular carcinomas were found to express BGIs. Why BGIs should be more readily detected in papillary tumours was not apparent.

The re-acquisition of A and B BGIs by some tumours may be taken as indirect evidence for the presence of a functional A and B glycosyl transferase. This contrasts with findings in the breast and prostate where antigen expression by malignant epithelium, as revealed by immunohistochemical techniques, appears limited to the re-expression of the H and Y isoantigens (Vowden *et al.*, 1986*a*, *b*). This may suggest a deficiency of A and B glycosyl transferases in these tumours.

The mechanism by which this change in antigen expression occurs has not been clearly defined. Hakomori (1981) has established that dramatic changes in cellular glycolipid composition and metabolism are associated with the oncogenic and ontogenic processes. Alternatively these changes may reflect alterations in the carbohydrate moieties of glycoproteins (Picard & Feizi, 1984). It would seem likely that variations in BGI expression represent a combination of these factors. It is equally clear that no one pattern of BGI expression exists. With malignant transformation the distal colon and thyroid show a partial re-acquisition of A, B and H BGIs, the breast and prostate while losing A and B BGIs totally, tend to retain H isoantigen and the stomach and urinary bladder shows a partial loss of all BGIs (Finan *et al.*, 1982, 1983). These findings would seem to offer some support to the suggestion that malignant cells may be regarded as cells held in some phase of their embryological development (Nowell, 1976). The pattern of malignant epithelial cell blood group antigen expression does show a remarkable parallel with those found by Szulman in his studies on embryological tissues (Szulman, 1960, 1962, 1964).

### References

COOPER, H.S., COX, J.B.A. & PATCHEFSKY, A.S. (1980). Immunological study of blood group substances in polyps of the distal colon. Expression of a fetal antigen. *Am. J. Clin. Path.*, **73**, 345.

COOPER, H.S. & HAESLER, W.E. (1978). Blood group substances as tumour antigens in the distal colon. *Am. J. Clin. Path.*, **69**, 594.

DAVIDSOHN, I. & STEJSKAL, R. (1972). Tissue antigens A, B and H in health and disease. *Haematologia*, **6**, 177.

DENK, H., TAPPEINER, G. & HOLZNER, J.H. (1974). Blood group substances (BG) as carcinofetal antigens in carinomas of the distal colon. *Eur. J. Cancer.*, **10**, 487.

DENK, H., HOLZNER, J.H. & OBIDITSCH-MAYR, I. (1975). Epithelial blood group antigens in colon polyps: Morphologic distribution and relationship to differentiation. *J. Natl Cancer Inst.*, **54**, 1313.

FINAN, P.J., ANDERSON, J.R., DOYLE, P.T., LENNOX, E.S. & BLEEHEN, N.M. (1982*a*). The prediction of invasive potential in superficial transitional cell carcinoma of the bladder. *Br. J. Urology.*, **54**, 720.

FINAN, P.J., ANDERSON, J.R., DOYLE, P.T., LENNOX, E.S. & BLEEHEN, N.M. (1982*b*). The prediction of invasive potential in superficial transitional cell carcinoma of the bladder. *Br. J. Urology*, **54**, 720.

FINAN, P.J., WRIGHT, D.G.D., LENNOX, E.S., SACKS, S.H. & BLEEHEN, N.M. (1983). Human blood group isoantigen expression on normal and malignant gastric epithelium studied with anti-A and anti-B monoclonal antibodies. *J. Natl Cancer Inst.*, **70**, 679.

FREDMAN, P., RICHERT, N.D., MAGNANI, J.L., WILLINGHAM, M.C., PASTAN, I. & GINSBURG, V. (1983). A monoclonal antibody that precipitates the glycoprotein receptor for epidermal growth factor is directed against the human group H Type 1 antigen. *Fed. Proc.*, **42**, 1988 (Abstract).

HAKOMORI, S. (1981). Glycosphingolipids in cellular interaction, differentiation and oncogenesis. *Ann. Rev. Biochem.*, **50**, 733.

HOLBOROW, E.J., BROWN, P.C., GLYNN, L.E., HAWES, M.D., GRESHAM, G.A., O'BRIEN, T.K. & COOMBS, R.R.A. (1960). The distribution of blood group A antigen in human tissues. *Br. J. Exp. Path.*, **41**, 430.

LLOYD, K.O., LARSON, G., STROMBERG, N., THURIN, J. & KARLSSON, K.A. (1983). Mouse monoclonal antibody F-3 recognizes the difucosyl Type 2 blood group structure. *Immunogenetics*, **17**, 537.

LOWE, A.D., LENNOX, E.S. & VOAK, D. (1983). A new monoclonal anti-A: culture supernatant with the performance of hyperimmune human reagents. *Vox. Sang.*, **46**, 29.

NOWELL, M.K. (1976). The clonal evaluation of tumour cell populations. *Science*, **194**, 23.

PICARD, J.K. & FEIZI, T. (1984). Carbohydrate antigens of the neoplastic and uninvolved mucosae of patients with carcinoma of the stomach and colon. *Biochem. Soc. Transact.*, **12**, 653.

RICHERT, N.D., WILLINGHAM, M.C. & PASTAN, I.H. (1983). Epidermal growth factor receptor: characterisation of a monoclonal antibody to the receptor of A431 cells. *Fed. Proc.*, **42**, 1904. (Abstract)

SZULMAN, A.E. (1960). The histological distribution of blood group substances A and B in man. *J. Exp. Med.*, **111**, 785.

SZULMAN, A.E. (1962). The histological distribution of blood group antigens in man as disclosed by immunofluoresence: II. The H antigen and its relationship to A and B antigens. *J. Exp. Med.*, **115**, 977.

SZULMAN, A.E. (1964). The histological distribution of blood group antigens in man as disclosed by immunofluorescence: III. The A, B and H antigens in embryos and foetuses from 18 mm in length. *J. Exp. Med.*, **119**, 503.

VOAK, D., LENNOX, E.S., SACKS, S., MILSTEIN, C. & DARNBOROUGH, J. (1982). Monoclonal anti-A and anti-B: Development as a cost-effective reagent. *Med. Lab. Sci.*, **39**, 109.

VOWDEN, P., LOWE, A.D., LENNOX, L.E. & BLEEHEN, N.M. (1984). Colonic polyp epithelial ABH blood group isoantigen (BGI) expression related to histological type and size. *Br. J. Surg.*, **71**, 906. (Abstract)

VOWDEN, P., LOWE, A.D., LENNOX, E.S. & BLEEHEN, N.M. (1986*a*). The expression of ABH and Y blood group antigens in benign and malignant breast tissue: The preservation of the H and Y antigens in malignant epithelium. *Br. J. Cancer*, **53**, 307.

VOWDEN, P., LOWE, A.D., LENNOX, E.S. & BLEEHEN, N.M. (1986*b*). Are blood group isoantigens lost from malignant prostatic epithelium? Immunohistochemical support for the preservation of the H isoantigen. *Br. J. Cancer*, **53**, 313.

WILEY, E.L., MENDESOHN, G. & EGGLESTON, J.C. (1981). Distribution of carcinoembryonic antigen and blood group substances in adenocarcinoma of the colon. *Lab. Invest.*, **44**, 507.